# Severe haze in northern China: A synergy of anthropogenic emissions and atmospheric processes

**DOI:** 10.1073/pnas.1900125116

**Published:** 2019-04-15

**Authors:** Zhisheng An, Ru-Jin Huang, Renyi Zhang, Xuexi Tie, Guohui Li, Junji Cao, Weijian Zhou, Zhengguo Shi, Yongming Han, Zhaolin Gu, Yuemeng Ji

**Affiliations:** ^a^State Key Laboratory of Loess and Quaternary Geology, Institute of Earth Environment, Chinese Academy of Sciences, Xi’an 710061, China;; ^b^Center for Excellence in Quaternary Science and Global Change, Chinese Academy of Sciences, Xi’an 710061, China;; ^c^Key Laboratory of Aerosol Chemistry and Physics, Institute of Earth Environment, Chinese Academy of Sciences, Xi’an 710061, China;; ^d^Interdisciplinary Research Center of Earth Science Frontier, Beijing Normal University, Beijing 100875, China;; ^e^Open Studio for Oceanic-Continental Climate and Environment Changes, Pilot National Laboratory for Marine Science and Technology (Qingdao), Qingdao 266061, China;; ^f^Department of Atmospheric Sciences, Texas A&M University, College Station, TX 77843;; ^g^Department of Chemistry, Texas A&M University, College Station, TX 77843;; ^h^Department of Earth and Environmental Sciences, Xi’an Jiaotong University, Xi’an 710049, China;; ^i^Guangzhou Key Laboratory of Environmental Catalysis and Pollution Control, School of Environmental Science and Engineering, Guangdong University of Technology, Guangzhou 510006, China

**Keywords:** severe haze, synergetic effects, anthropogenic emission, atmospheric chemistry, climate change

## Abstract

Regional severe haze represents an enormous environmental problem in China, influencing air quality, human health, ecosystem, weather, and climate. These extremes are characterized by exceedingly high concentrations of fine particulate matter (smaller than 2.5 µm, or PM_2.5_) and occur with extensive temporal (on a daily, weekly, to monthly timescale) and spatial (over a million square kilometers) coverage. Although significant advances have been made in field measurements, model simulations, and laboratory experiments for fine PM over recent years, the causes for severe haze formation have not yet to be systematically/comprehensively evaluated. This review provides a synthetic synopsis of recent advances in understanding the fundamental mechanisms of severe haze formation in northern China, focusing on emission sources, chemical formation and transformation, and meteorological and climatic conditions. In particular, we highlight the synergetic effects from the interactions between anthropogenic emissions and atmospheric processes. Current challenges and future research directions to improve the understanding of severe haze pollution as well as plausible regulatory implications on a scientific basis are also discussed.

## Significance

Severe haze events with large temporal/spatial coverages have occurred frequently in wintertime northern China. These extremes result from a complex interplay between emissions and atmospheric processes and provide a unique scientific platform to gain insights into many aspects of the relevant atmospheric chemistry and physics. Here we synthesize recent progress in understanding severe haze formation in northern China. In particular, we highlight that improved understanding of the emission sources, physical/chemical processes during haze evolution, and interactions with meteorological/climatic changes are necessary to unravel the causes, mechanisms, and trends for haze pollution. This viewpoint established on the basis of sound science is critical for improving haze prediction/forecast, formulating effective regulatory policies by decision makers, and raising public awareness of environmental protection.

Rapid industrialization/urbanization in developing countries has resulted in increased air pollution, along a trajectory similar to that previously encountered in many developed nations. As the world’s largest developing country, China has experienced haze pollution over the recent decades (1–3), which is defined as a weather phenomenon with a horizontal visibility of less than 10 km due to dense accumulation of fine particulate matter (particles with an aerodynamic diameter smaller than 2.5 μm, or PM_2.5_) (4). PM is emitted directly into the atmosphere (referred to as primary particles) or produced in the atmosphere via gas-to-particle conversion (referred to as secondary particles) (5–7). In addition, primary and secondary PM undergo chemical and physical transformations and are subjected to cloud processing and removal from air (5, 6). Severe haze events with exceedingly high PM mass loading (from 100 to 1,000 μg⋅m^−3^) and large temporal/spatial coverages have occurred persistently in northern China, particularly in the North China Plain (NCP). Noticeably, a haze extreme occurred in January 2013 that lasted close to 1 mo and affected a total area of ∼1.3 million km^2^ and ∼800 million people.

High emissions of primary particles and gaseous PM precursors from multiple sources, efficient secondary PM formation, regional transport, adverse meteorological and climatic conditions, and their synergetic effects have been identified as the main factors regulating the frequency and severity of haze formation in northern China (8–15). Noticeably, high levels of PM gas precursors lead to significant production of secondary PM, as documented by large fractions and high abundances of secondary organic aerosol (SOA) and secondary inorganic aerosol (SIA) from field measurements (8, 9, 12, 16). Also, PM accumulation and secondary formation are enhanced under stagnant meteorological conditions, characterized by high relative humidity (RH), low planetary boundary layer (PBL) height, and weak surface winds (12, 17). In addition, climate change represents another plausible factor influencing haze formation in the NCP (18).

The wintertime daily average mass concentrations of PM_2.5_ in many major cities in northern China are typically one to two orders of magnitude higher than those in urban areas of the United States and Europe (19, 20). Elevated PM levels are often accompanied by a sharp increase in respiratory diseases (21). Long-term exposure to high levels of PM_2.5_ is estimated to have resulted in 1.1 million deaths in 2015 in China (22). Also, aerosols absorb and scatter solar radiation, leading to important consequences for atmospheric stability and energy budget. Such an effect, commonly referred to as an aerosol-radiation interaction (ARI), contributes importantly to cooling (by scattering and absorption) at the surface and warming (by absorption) in the atmosphere (23–25). An increased atmospheric stability due to the ARI effect exacerbates the formation and accumulation of fine PM (25, 26). By serving as cloud condensation nuclei (CCN) and ice nucleating particles, aerosols influence the macro- and microphysical properties of clouds (27, 28). This latter effect, often referred to as an aerosol–cloud interaction (ACI), modifies the lifetime and albedo of clouds and precipitation efficiency (29, 30) and weakens the monsoon circulations (31, 32). The chemical and physical transformations of both primary and secondary PM further complicate the physicochemical properties of fine particles, including the optical and hygroscopic properties that impact their lifetimes, cloud formation potential, and radiative forcing (5, 6).

The haze extremes in China have provided a unique scientific platform to gain insights into many aspects of the relevant atmospheric chemical and physical processes (i.e., formation, transformation, transport, and removal of PM), and considerable scientific advances have been made in understanding PM pollution and its interactions with atmospheric processes (8, 9, 11, 12, 14, 15, 17, 33–37). Several previous reviews have focused on different aspects of the formation, chemical characteristics, and control strategies for haze pollution in China (3, 5, 38–40). In this paper, we synthesize recent progress in understanding the fundamental aspects of severe haze pollution in northern China and discuss the current challenges, future research directions, and plausible regulatory implications on a scientific basis.

## A Historical Perspective

Distinct from the historic 1952 London fog mainly caused by coal combustion and the Los Angeles smog mainly caused by photochemical oxidation of vehicular emissions (5, 41), haze pollution in China is the consequence of diverse, high primary emissions and efficient secondary production (8, 9, 16, 17, 37, 42, 43). In addition, regional transport of pollutants and emissions on a broader geographical scale further aggravates severe haze formation (44), leading to significant challenges in PM source characterization.

**Fig. 1.**
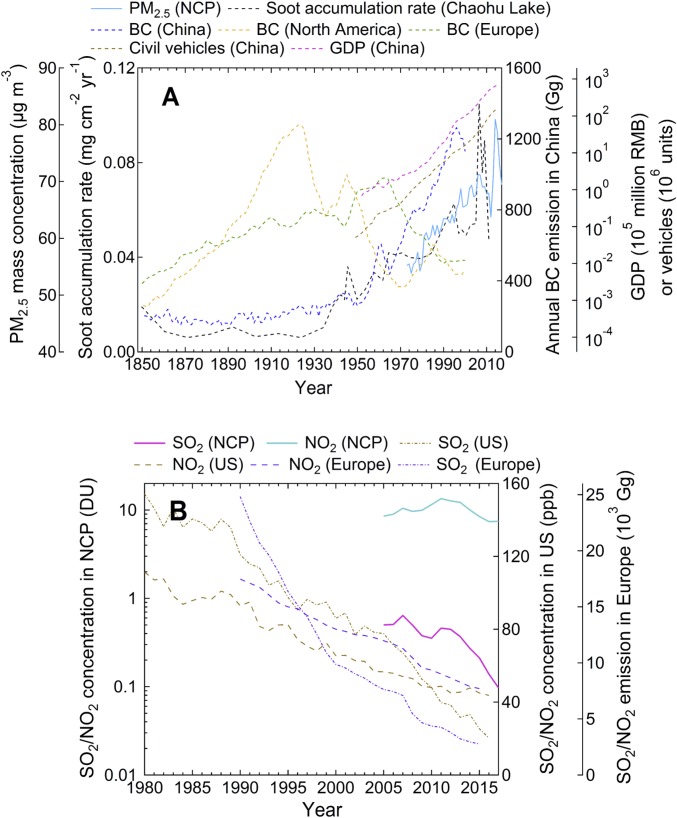
The historical variations of PM_2.5_ in the NCP, soot accumulation in Chaohu Lake, civil vehicles and gross domestic product (GDP) in China, and BC emissions in China, North America, and Europe from 1850 to 2017 (*A*) and the concentrations of SO_2_ and NO_2_ in the NCP, United States, and Europe over the past three decades (*B*). The PM_2.5_ was retrieved from visibility data and the soot record from Chaohu Lake, China (46); the BC emissions in China, North America, and Europe were derived from emission inventory of Bond et al. (47); the concentrations of SO_2_ and NO_2_ in the NCP were retrieved from Aura OMI satellite data (in unit of Dobson Unit, DU); the concentrations of SO_2_ and NO_2_ in the United States were obtained from the US EPA (https://www.epa.gov/criteria-airpollutants); and the emissions of SO_2_ and NO_2_ in Europe were derived from EMEP emission database (http://www.emep.int). The civil vehicles and GDP were obtained from China Statistical Yearbook (www.stats.gov.cn). Note that the trough of PM_2.5_ from 2007 to 2012 could be attributed to the implementation of the “Energy Conservation and Emissions Reduction” policy from 2006 (*SI Appendix*), while the latest decrease of PM_2.5_ from 2014 to 2017 could be attributed to the implementation of the “Air Pollution Prevention and Control Action Plan” from 2013.

The annual PM_2.5_ concentrations in the NCP, reconstructed from daily visibility data from 20 observatory stations (*SI Appendix*, Figs. S1 and S2), exhibit an increasing trend over the past four decades (Fig. 1*A*), much higher than the annual standard of 10 μg⋅m^−3^ established by the World Health Organization. This trend of increasing PM_2.5_ coincides with a continuous growth in the gross domestic product (GDP), energy consumption, and vehicular fleets in China. For example, the GDP in this region increased from 113 billion Chinese Yuan (¥) in 1978 to 15,978 billion ¥ in 2010, and the consumption of coal and crude oil increased from 363 and 72 to 1,348 and 140 million tons of standard coal equivalent from 1998 to 2010, respectively (45).

There is also an increasing trend of black carbon (BC) concentrations in China (46), particularly after the 1970s, in sharp contrast to the decreasing trend of BC in North America since the 1920s and in Europe since the 1960s (Fig. 1*A*) (47). The SO_2_ concentration in the NCP has only declined recently (Fig. 1*B*), in contrast to considerably declining SO_2_ and NO_2_ in the United States and Europe over the past decades. NO_x_ emissions in China increased significantly from 11.0 megatons (Mt) in 1995 to 26.1 Mt in 2010 and are projected to increase by 36% by 2030 relative to 2010 (48). Agricultural activities in China are estimated to have contributed ∼3.2 Tg of NH_3_ from fertilizer application and ∼5.3 Tg of NH_3_ from livestock in 2006 (49). Vehicular emissions are likely another important urban source of NH_3_ (50–52), although this vehicular NH_3_ source is still debatable (53).

The haze extremes since January 2013 have prompted a variety of legislative actions from the central to local governments for improvement of air quality in China, including a legislative “Air Pollution Prevention and Control Action Plan” established in 2013, a regulatory “Air Pollution Prevention and Control Law” implemented in 2016, and a number of short-term regulatory measures to reduce pollutant emissions, for example the “odd–even vehicular ban” experiments and temporary industrial shut-down in many cities. In addition, the Chinese central and local governments have undertaken major efforts to improve the scientific understanding of haze pollution, especially in the Beijing–Tianjin–Hebei (BTH) region and Fenwei Plain, including large projects for intensive field measurements for “2+26 cities” in BTH and “11 cities” in the Fenwei Plain. Such large-scale atmospheric field campaigns are aimed at improving the capability of haze forecast and providing policy makers with scientifically based control strategies to mitigate haze pollution.

The haze episodes in northern China have become more frequent and severe in recent decades (1, 18, 19), and a variety of regulatory measures have been implemented to improve air quality by Chinese central and local governments. Noticeably, these regulatory measures have resulted in significant reduction in primary PM emissions from industry and other sources (54, 55), but emissions of gas precursors for secondary aerosols, including SO_2_, NO_x_, NH_3_, and volatile organic compounds (VOCs), remain at high levels and contribute to high abundances of secondary PM, most noticeably SOA and SIA (8, 9, 17).

## Geographic and Meteorological Characteristics

**Fig. 2.**
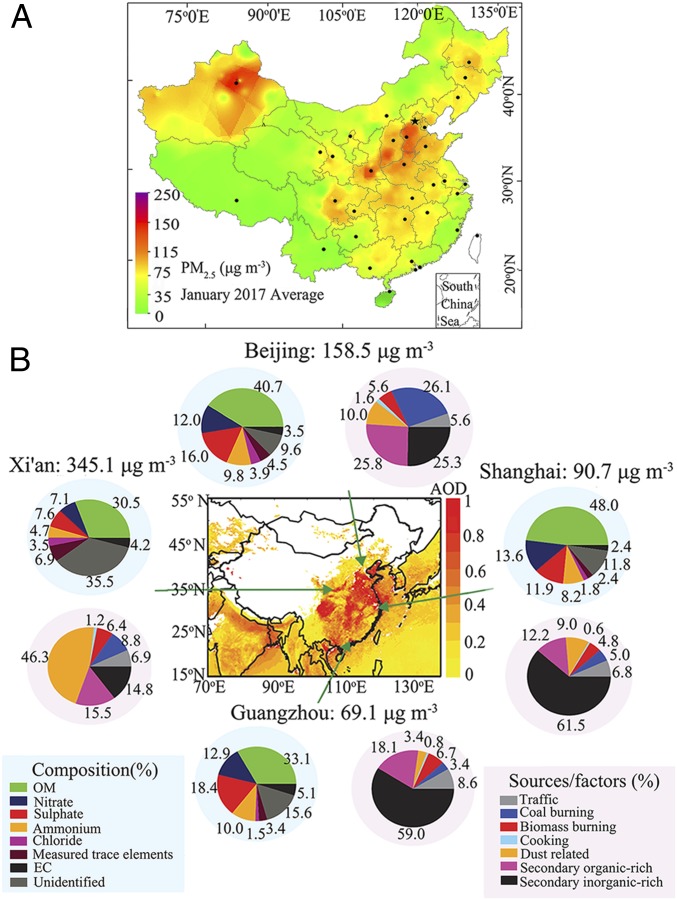
Average concentration of PM_2.5_ in January 2017 measured from nationwide ground stations, obtained from the China National Environmental Monitoring Center (*A*) and the chemical composition and sources of PM_2.5_ in four representative megacities during the severe haze pollution events in January 2013 (*B*) (reprinted from ref. 8).

Haze pollution in northern China typically encompasses a large geographic area, from offshore eastern China (125° E) to western China (100° E). In particular, the NCP, Fenwei Plain, and Chengdu-Chongqing Plain have suffered from severe haze pollution (Fig. 2). In addition to the haze extreme in January 2013, two large-scale severe haze episodes in northern China reached the “red alarm” stage (the highest air-quality warning level in China) during the winter of 2016/2017. Large-scale haze pollution in the NCP, which was often observed as widespread haze plumes over the entire region, has been linked to orographic forcing (2, 56). The NCP region is surrounded by the Yan Mountains to the north, the Taihang Mountains and the Loess Plateau to the west, and the Bohai Sea to the east. The southern part of the NCP is flat with dense populations and industrial facilities. Such a unique basin terrain is unfavorable for pollutant dispersion and is highly susceptible to stagnation development and regional transport, leading to accumulation of air pollutants.

Haze pollution in northern China occurs in all seasons and is most frequent and severe in winter (1, 2). Winter meteorological conditions in this region are mainly controlled by synoptic weather patterns. For example, wind circulation in the NCP typically exhibits a periodic cycle of 4 to 7 d, which is also correlated with a similar cycle in the PM_2.5_ concentrations from the clean, transition, to polluted periods (9). The periodic haze cycles are evident from large variations of several meteorological parameters, characteristic of stagnant development and accumulation/secondary formation of fine PM (2, 8, 9, 17, 33, 37). Strong northwesterly/northeasterly (>4 m⋅s^−1^) wind from less populated mountainous areas and low RH (<40%) are most frequent during the clean period (PM_2.5_ < 25 μg⋅m^−3^). During the transition period, the prevailing winds switch from northerly to southerly with a considerably decreased speed, and the PM_2.5_ concentration increases rapidly at a rate of some micrograms per cubic meter per hour. Weak southerly wind from heavily populated, industrial regions and high RH (*SI Appendix*, Fig. S3) are most prevalent during the polluted period, which spans from 1 d to over half a month, depending on the meteorological conditions. Long-term measurements in Beijing from April 2013 to December 2017 showed that severe haze events mainly occur with prevailing southerly winds of less than 3 m⋅s^−1^ (*SI Appendix*, Fig. S4), when the daily PM_2.5_ concentration often remains several times higher than the national standard of 75 μg⋅m^−3^ (three times that of the WHO standard of 25 μg⋅m^−3^), and the hourly PM_2.5_ concentration even exceeds 1,000 μg⋅m^−3^. In January 2017, for example, the average PM_2.5_ concentrations in the NCP and Fenwei Plain were higher than 115 μg⋅m^−3^ (Fig. 2*A*).

## Primary PM Source

Urban fine PM consists of a highly complex mixture of inorganic and organic aerosol (OA) produced from a wide variety of natural and anthropogenic sources (5, 6). OA, water-soluble inorganic ions (e.g., NH_4_^+^, NO_3_^−^, and SO_4_^2−^), and mineral dust are the dominant PM constituents (19, 57). OA comprises as high as more than 50% of the total mass of fine PM during haze events, and its formation is less understood than that of the inorganic fraction (8, 9, 33, 37, 58, 59). OA is broadly classified as primary OA (POA) directly emitted or SOA formed in the atmosphere.

Source apportionment studies have identified several major primary sources of fine PM in northern China, including traffic, coal combustion, biomass burning, cooking, and dust (8, 16, 33, 37, 58, 59). A major difference in the primary emissions of this region from those in the United States and European countries is the source of residential coal combustion related to cooking and wintertime heating. Coal combustion constituted 26% of the PM_2.5_ mass during the January 2013 severe haze event (8) and 33% of the OA mass from November 2011 to January 2012 in Beijing (42). The average contributions of traffic to the OA vary from 9 to 18%, while cooking emissions vary from 6 to 32% of OA (33, 42, 60, 61). High fugitive dust emission represents another feature in the arid and semiarid regions in northwest China. For example, dust constituted 46% of the PM_2.5_ mass in Xi’an during the severe event in January 2013 (8). The presence of large amounts of metal (e.g., transition metal ions) in dust particles likely promotes catalytic reactions for secondary aerosol formation, for example sulfate formation through iron catalytic reactions (10). In addition, there exists a noticeable geographical difference of the emission sources in northern China. For example, during the wintertime heating period, coal combustion is the main emission source in Beijing and its surrounding areas, while biomass burning represents the main contributor in Xi’an (33).

## Secondary PM Formation and Transformation

**Fig. 3.**
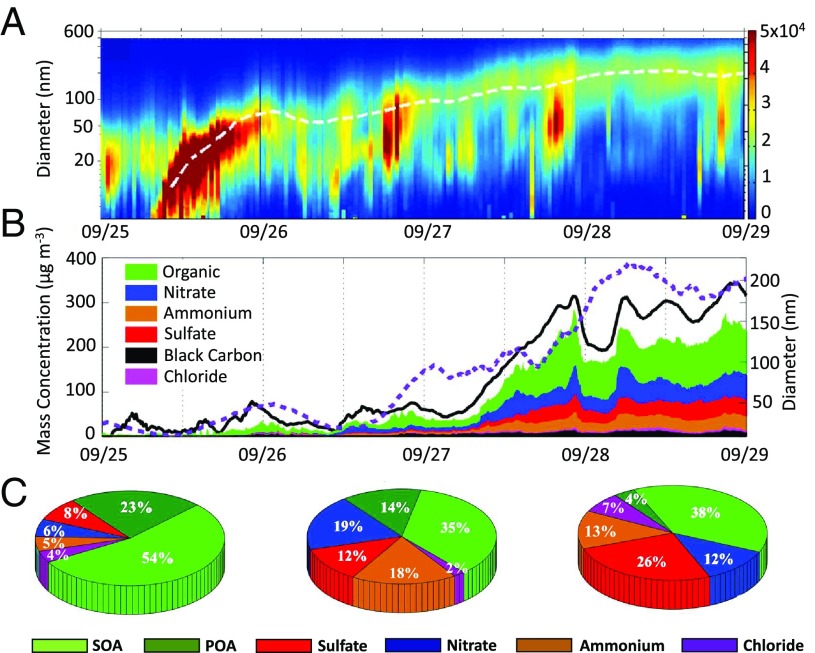
Aerosol nucleation and growth during a pollution episode (September 25–29, 2013) in Beijing. Temporal evolutions of particle number size distribution and mean diameter (white dashed curve) (*A*); PM_2.5_ mass concentration (black solid line), mean diameter (purple dashed line), and PM_1_ (particulate matter smaller than 1.0 μm) chemical composition (*B*); evolution of particle chemical composition from clean (*Left*), transition (*Middle*), to polluted (*Right*) periods (*C*). Modified from ref. 9.

Fine PM typically consists of large fractions of secondary aerosols, including SOA and SIA (Fig. 2*B*). Huang et al. (8) found that the severe haze events are driven to a large extent by secondary aerosol formation and the contribution from SOA is as significant as that from SIA. The formation and transformation of secondary aerosols involve several complex chemical processes, including photochemical oxidation, nucleation, condensation/partitioning, heterogeneous reactions, and nocturnal reactions (5, 6). The periodic cycle in the PM_2.5_ mass concentration is also evident with the physical and chemical transformation of PM, including the size, number concentration, chemical composition, effective density, hygroscopicity, and optical properties (9, 17, 33). During haze evolution, particles grow to much larger sizes. For example, the particles peaked at about 800 nm during haze extremes in Xi’an and Beijing, about twice that during less polluted episodes (33). Fig. 3 illustrates the transformation of particle size and chemical composition from clean, transition, to polluted periods, which consequently affects the effective density and hygroscopicity (*SI Appendix*, Fig. S5). Furthermore, light scattering and absorption by PM increase during haze development. Those variations in the particle properties are indicative of distinct formation, growth, and transformation of PM. Noticeably, the contribution of secondary PM in northern China is expected to continuously increase because of diverse sources and high emissions of PM precursor gases (62), in contrast to the significant decline in primary PM emissions that resulted from the strict regulatory controls.

### New Particle Formation.

New particle formation (NPF) events have been frequently observed in different urban areas of China (5, 9, 63–65). The presence of high concentrations of nucleation precursor gases including SO_2_, NH_3_, amines, and VOCs likely accounts for the nucleation and growth of nanoparticles in China (5, 65). NPF is typically limited by thermodynamic and kinetic barriers (i.e., the nucleation barrier relevant to the free energy and the Kelvin barrier relevant to the particle curvature, respectively) (5). NPF and its subsequent growth has been shown to increase the CCN concentration (66), for example, by a factor of 5.6 to 8.7 in the NCP (63). NPF represents an important source of ultrafine particles (with an aerodynamic diameter of less than 0.1 μm, UFPs) during the early stage of haze pollution events (5, 9). The NPF mechanisms in urban environments remain uncertain, particularly in terms of identification of the chemical species responsible for the nucleation and growth of nanoparticles.

### Formation of SIA.

The importance of SO_4_^2−^, NO_3_^−^, and NH_4_^+^ in severe haze formation in China has been documented in many field studies (8, 9, 17, 33, 37, 67, 68). The rapid increase of sulfate at high RH has been observed at many sites in China, suggesting that aqueous-phase oxidation of SO_2_ plays an important role during severe haze events (10, 33, 37, 69). The gas-phase oxidation of SO_2_ by OH is a slow process, with a corresponding SO_2_ lifetime of 5 to 10 d (5). Several aqueous pathways of SO_2_ oxidation have been proposed, including its reactions with dissolved ozone, hydrogen peroxide, organic peroxides, NO_2_, and OH via catalytic or noncatalytic pathways involving mineral oxides (5). Model simulations showed that sulfate heterogeneous formation from SO_2_ with enough alkalinity to maintain the high iron-catalyzed reaction rate substantially improves the sulfate simulations compared with the measurements in Xi’an and Beijing (10).

A recent laboratory/field study of winter haze events in Beijing and Xi’an found that the aqueous oxidation of SO_2_ by NO_2_ is key to efficient sulfate formation under the conditions of high RH and NH_3_ neutralization or under cloud conditions (17). Wang et al. (17) showed that organic seed particles exposed simultaneously to SO_2_, NO_2_, and NH_3_ exhibit significant sulfate production at high RH and derived an experimental uptake coefficient (8.3 ± 5.7 × 10^−5^) consistent with that (4.5 ± 1.1 × 10^−5^) from field measurements in Beijing. Another modeling study suggested a self-amplifying mechanism for sulfate production involving reactive nitrogen chemistry (34). Using a thermodynamic model, Cheng et al. (34) determined an aerosol pH ranging from 5.4 to 6.2 during haze periods in Beijing and concluded that the aqueous oxidation pathway of SO_2_ by NO_2_ represents the missing source for sulfate production in their modeling simulations. However, using a similar thermodynamic approach (i.e., treating the PM exclusively as a mixture of inorganic salts dominated by ammonium sulfate and neglecting the effects of organic compounds), Guo et al. (70) found that the particle pH is always acidic, even for the unusually high NH_3_ levels in Beijing (pH = 4.5) and Xi’an (pH = 5) and concluded a negligible role of aqueous oxidation SO_2_ by NO_2_ in China. Most recently, Wang et al. (71) showed distinct size growth and sulfate formation between ammonium sulfate and oxalic acid seed particles exposed to vapors of SO_2_, NO_2_, and NH_3_ at high RH, indicating that a particle mixture of inorganic salts adopted by the previous studies does not represent a suitable model system and that the acidity and sulfate formation cannot be reliably inferred without accounting for the effects of multiaerosol compositions (i.e., organics) during severe haze events in China. Noticeably, current estimation of the pH values using the thermodynamic models is highly uncertain, because of several intricate difficulties, such as the applicability of the thermodynamic model to nonequilibrium conditions, the phase (e.g., liquid, amorphous, or crystalline) and mixing states of PM, the acid–base reactions involving organic acids and NH_3_/amines, and the presence of multicomponent organic and inorganic species (5, 71–73). Furthermore, available kinetic experiments of the aqueous oxidation of SO_2_ by NO_2_ did not account for the gaseous uptake process and differed by over two orders of magnitude (74, 75). Clearly, elucidation of the sulfate formation mechanism during severe haze in China awaits direct measurement of the particle acidity to validate the thermodynamic models and refined laboratory kinetic experiments of aqueous SO_2_ oxidation under atmospheric conditions.

Gas-phase oxidation of NO_2_ by OH (76, 77) and the heterogeneous reaction of N_2_O_5_ (78–80) represent the important pathways for nitrate formation during day and night, respectively. HNO_3_ and ammonium nitrate (NH_4_NO_3_) are both subject to thermodynamic partitioning between the gas and particle phases (7, 81), because of their high volatility and thermal stability, respectively. Low temperature and high RH facilitate the gas-to-particle partitioning, explaining the high nitrate concentration during haze events in winter. Also, SO_4_^2−^ likely competes with NO_3_^−^ for NH_4_^+^ in PM formation. Under NH_3_-deficient conditions, alkaline metals (e.g., Ca^2+^, Mg^2+^, and K^+^) also participate in the competing neutralization of sulfate and nitrate, which is relevant to northern China due to the presence of dust particles.

**Fig. 4.**
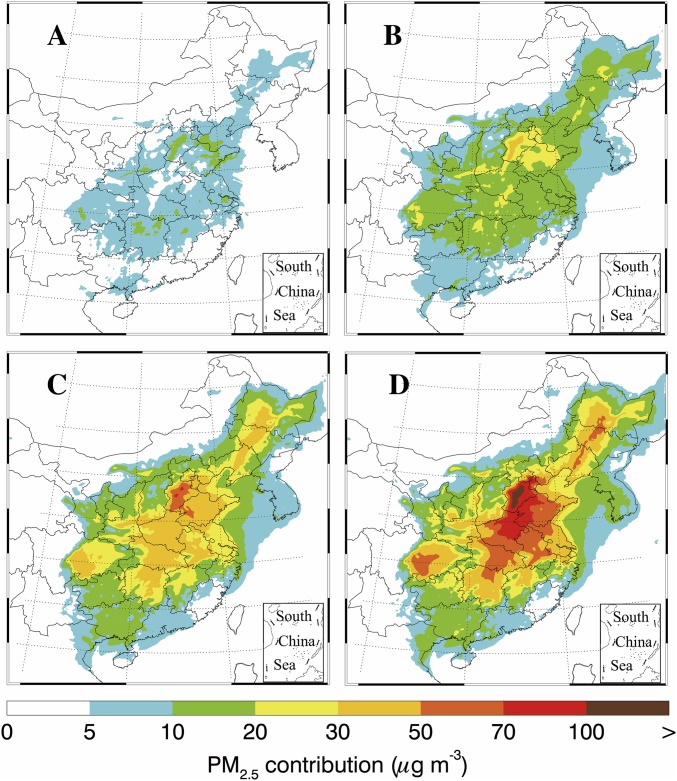
Contribution of agricultural NH_3_ associated reactions to PM_2.5_ mass, simulated by the WRF-Chem model in four different scenarios (*A–D*, representing 25, 50, 75, and 100% of the current NH_3_ level, respectively).

Elevated NH_3_ concentrations have been observed during severe haze events in northern China (17). For example, the annual average of NH_3_ at an urban site in Beijing was 18.5 ± 13.8 ppb in 2008 and 23.5 ± 18.0 ppb in 2009 (50), much higher than that in urban air in the United States and Europe (typically <5 ppb) (82, 83). In contrast to those in developed countries, agricultural NH_3_ emissions largely overlap with the industrial emissions of SO_2_ and NO_2_ in northern China (*SI Appendix*, Fig. S6). Such an overlap considerably favors the formation of NH_4_^+^, SO_4_^2−^, and NO_3_^−^. A model study using WRF-Chem (see *SI Appendix* for more details) showed that the average contribution of the agricultural NH_3_ emissions in the NCP was ∼30% of the PM_2.5_ mass (or ∼42 μg⋅m^−3^) during a severe haze event in December 2015 (Fig. 4); a 50% reduction in the NH_3_ concentration yields 10% reduction in PM_2.5_ (or ∼13 μg⋅m^−3^). Considering the significance of NH_3_, some important questions need to be further assessed. For example, can severe haze formation be effectively prevented by significant reduction in emissions of agricultural NH_3_ to interfere with the formation of SIA and SOA, and will pollution disasters similar to the 1952 London fog occur in China if only agricultural NH_3_ emission is significantly reduced, while emissions of SO_2_ and NO_2_ remain high, leading to highly acidic haze particles and therefore adverse health effects?

### Formation of SOA.

In contrast to SIA that has a single precursor (i.e., SO_2_, NO_x_, or NH_3_), SOA is produced from oxidation of a large variety of VOCs with distinct functionality, reactivity, product distributions, and PM formation potential (5, 6, 84). The chemical complexity of VOCs, together with large spatial and temporal variability in their sources and the atmospheric oxidative capacity, renders significant challenges in quantitative understanding of SOA formation. A current view of SOA formation is that this process is dominated by equilibrium partitioning between the gas and particle phases for the products of VOC oxidation reactions, which is dependent on the volatility (i.e., from semi-, intermediate-, low- to extremely low-volatility organics) (85). Multiphase chemistry and particle-phase oligomerization also likely contribute to SOA formation. Atmospheric models, however, have consistently underestimated the SOA mass measured in field studies, and such an inconsistency is likely attributed to missing emissions/precursors or inaccurate/unaccounted chemistry (84, 86). The latter topic has received close attention because of increasing evidence from laboratory/field studies showing that heterogeneous reactions of oxygenated organics, such as small α-dicarbonyls and aldehydes, produce multifunctional, higher-molecular-weight oligomers/polymers with low saturation vapor pressures and shift the physical partitioning to increase SOA yields (84, 87, 88).

The emissions and oxidation for VOCs are still not well understood, hindering quantitative assessment of SOA formation (5, 84, 86). For example, recent studies showed distinct mechanisms leading to SOA formation from acetylacetone and toluene (89, 90). In particular, the aromatic chemistry needs to be improved to realistically predict SOA formation in atmospheric models, since aromatics correspond to the most abundant VOCs and SOA precursors under the urban environments. Smog chamber studies on the aging of biomass burning emissions by Huang et al. (8) illustrated clearly that SOA is efficiently produced (exceeding POA) at OH concentrations typical of wintertime conditions in China. Ambient measurements in northern China showed that when the odd oxygen concentrations (O_x_ = O_3_ + NO_2_) are high (e.g., >80 μg⋅m^−3^) the SOA fraction correlates positively with O_x_, suggesting the significance of atmospheric photochemical processes in SOA formation (16, 91).

Wang et al. (17) indicated that there exists a transition from photochemical to aqueous production of SOA formation during the haze evolution in China; photochemistry dominates in the early stage of haze development, but aqueous chemistry dominates during the transition and polluted periods. Also, the aqueous productions of SOA and SIA likely mutually enhance each other; such an effect is clearly evident from concurrently large increases in their mass concentrations at high RH, when the photochemical activity is considerably decreased because of weak UV during winter (9, 17). Less-oxidized SOA (LO-OOA) has been shown to play a more important role during the early stage of haze episodes (91). Measurements in winter 2013/2014 in Beijing showed that the aqueous-phase reactions likely play an important role in the formation of more-oxidized SOA (MO-OOA) during the polluted period, since RH or aerosol liquid water content (ALWC) exhibits an obvious effect on the MO-OOA contribution to total OA (91). The aqueous-phase formation of MO-OOA also alters the degree of SOA oxidation, as reflected by high O/C ratios at high RH. Also, measurements in winter 2010 in Beijing indicated that LO-OOA formation may be driven by aqueous or cloud chemistry (67). By comparing high- and low-RH events of similar PM levels, Huang et al. (37) found that SOA and SIA dominate at high RH, but POA dominates at low RH, highlighting the importance of aqueous-phase chemistry in SOA formation. The nighttime NO_3_ chemistry represents another possible pathway contributing to SOA production (92). Also, heterogeneous conversion of NO_2_ to HONO and its subsequent photolysis likely constitute an important source for OH (17, 93, 94) and play a key role in VOC oxidation during the polluted period.

## Interactions Among Different Atmospheric Processes

### Effects of ARI and ACI.

There exists a strong interaction between the PM level and atmospheric stability, relevant to the ARI (25). A haze layer cools the Earth’s surface by scattering and absorbing and heats the atmosphere by absorbing solar radiation (*SI Appendix*, Fig. S7). The ARI effect increases the air static stability and results in PM accumulation at the lower troposphere. Severe haze formation is clearly linked to unfavorable meteorological conditions (i.e., shallow PBL, low wind speeds, and high RH) (2, 9, 17, 95, 96). In addition, high RH enhances ALWC and facilitates aqueous formation of SOA and SIA (16, 17, 96). Ambient measurements in Beijing showed that the SIA fraction in PM_2.5_ (24 to 55%) increases with increasing RH (15 to 83%), indicating a feedback mechanism relevant to equilibrium partitioning and aqueous chemistry (97). The simultaneously elevated RH levels and SIA mass concentrations result in an abundant ALWC, which acts as an efficient medium for multiphase reactions and accelerates severe haze formation (97). ALWC, together with aerosol chemical composition, scatters or absorbs a fraction of the incoming solar radiation to cool or warm the atmosphere, decreasing surface temperature and altering atmospheric stability. In addition, the multiphase reactions of VOCs yield multifunctional, light-absorbing products (referred to as brown carbon or BrC; ref. 98). For example, the heterogeneous reactions between small α-dicarbonyls (glyoxal and methylglyoxal) and base species (NH_3_ or amines) form N-heterocycles that absorb both UV and visible radiation (99, 100). Furthermore, SOA has been identified as key species responsible for aging and transformation of BC particles; rapid coating by organics on BC not only leads to large morphology variation but also significantly impacts the BC lifetimes and ARI because of enhanced light absorption and scattering (24, 101). Hence, the ARI effects due to BrC and SOA as well as their interactions with BC may play a key role in the development of stagnation in northern China. In addition, the aerosol single-scattering albedo (SSA) determines the scattering and absorption of solar radiation by aerosols and the diffuse radiation reaching the ground surface, which influence the atmospheric stability and net primary productivity (102). Field measurements have shown high light absorption capability of haze aerosols in the NCP, with the derived SSA of ∼0.9 (103–105). The absorption by BC has been proposed to decrease the PBL height by up to 15% during winter haze events (106).

Field studies showed that high PM_2.5_ concentrations increase the air stability due to the ARI, leading to decreased PBL height (15, 36, 96, 107, 108). Zhang et al. (36) illustrated clearly that the increase in aerosol pollution from the ground can lead to surface cooling by ARI, which facilitates temperature inversions, increases moisture accumulations, and results in extra deterioration of meteorological conditions. A positive feedback cycle involving the interaction between PM, PBL, and water vapor constitutes a self-amplification mechanism to trap PM near the surface. Under stagnant meteorological conditions in winter, the dispersal of water vapor is constrained by a shallow PBL, leading to an increase in RH. An increasing RH promotes aerosol hygroscopic growth and multiphase reactions and augments the particle size and mass, leading to further dimming and decreases of the surface temperature and PBL height, therefore enhancing the surface aerosol concentrations and RH (*SI Appendix*, Fig. S8) (96). Coupled chemical transport models quantified the impacts of ARI on the PBL height and surface PM_2.5_ concentrations during severe haze episodes in the NCP (109, 110). Elevated levels of PM_2.5_ attenuate solar radiation at the surface, cool the lower troposphere, and hinder PM_2.5_ dispersion. The PBL height decreases linearly with increasing PM_2.5_ concentration due to the ARI (*SI Appendix*, Fig. S9*A*). Furthermore, the near-surface PM_2.5_ concentration of less than 75 μg⋅m^−3^ corresponds to smaller ARI and resultant perturbation of wind fields. However, the near-surface PM_2.5_ mass concentration from 75 μg⋅m^−3^ to several hundred micrograms per cubic meter is resulted from a larger ARI, enhancing PM_2.5_ by up to 25% in the NCP (*SI Appendix*, Fig. S9*B*).

### Impacts of Climate Change.

**Fig. 5.**
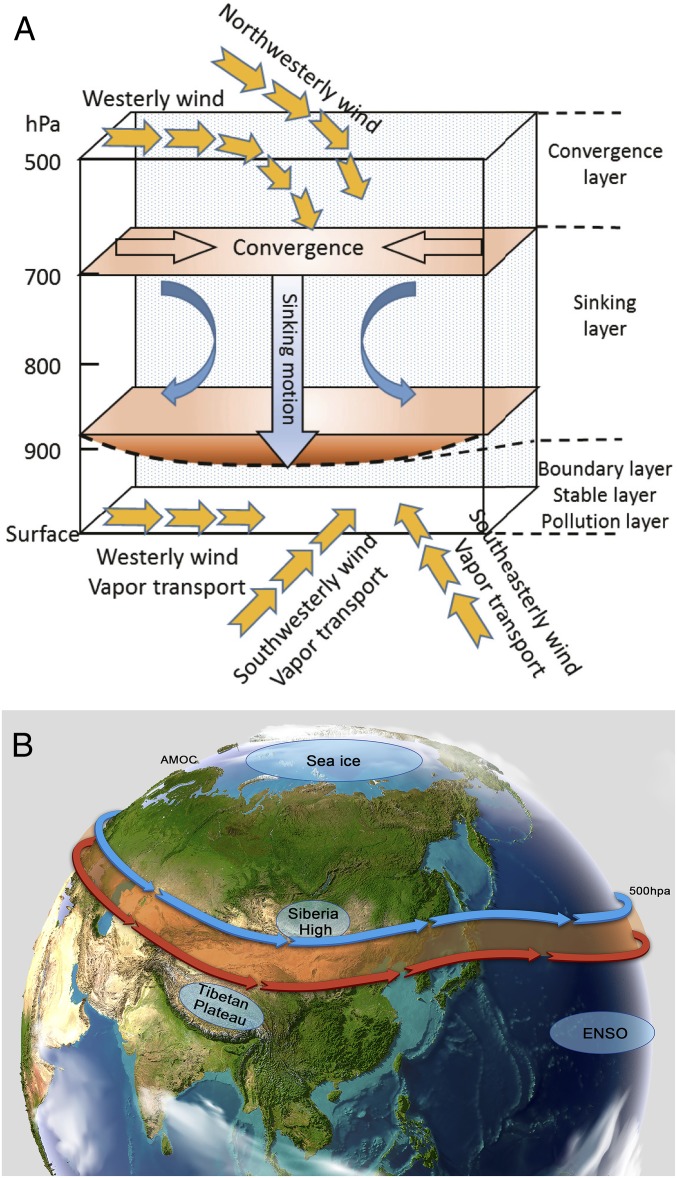
Schematic representation of the synoptic background that facilitates the severe haze formation (*A*) (reprinted from ref. 115) and those climatic factors affecting the regional East Asian winter monsoon and westerly circulation, including the variations of Arctic sea ice and Siberian High, topography of Tibetan Plateau, ENSO, and AMOC (*B*).

The meteorological conditions in northern China are determined by synoptic-scale weather patterns and further modulated by local circulations. Climate change influences several aspects of the large-scale circulations in this region, which ultimately regulates the transport and dispersion of pollutants (18, 111, 112). Under weak large-scale wind conditions, local circulations, such as land–sea breeze or mountain–valley breeze, become the predominant factor in controlling pollution transport (113). When northern China is controlled by near zonal westerly airflow or northwesterly airflow in the midupper troposphere, descending motion in the midlower troposphere is induced, reducing the PBL height and causing an inversion in the lower troposphere (114). The inversion causes air pollutants and moisture to progressively accumulate in the lower PBL, facilitating severe haze formation (Fig. 5*A*) (115). Severe haze events are usually weakened or disappear with the outbreak of northern cold air. The occurrence of westerly or northwesterly airflow over northern China is mainly controlled by the regional East Asian winter monsoon and westerly circulation, which is further influenced, particularly under the framework of global warming, by various factors including variations of Arctic sea ice and Siberia High, topography of the Tibetan Plateau, El Niño and Southern Oscillation (ENSO), and potentially the Atlantic meridional overturning circulation (AMOC) (Fig. 5*B*).

Global warming associated with increasing greenhouse gases in the atmosphere affects the frequency and severity of haze formation by modulating weather conditions. Zhang et al. (36) pointed out that the decadal worsening of meteorological conditions can partly be attributed to climate warming. Cai et al. (18) found that with future global warming (2050 to 2099) the frequency of unfavorable weather conditions for severe haze events similar to that in January 2013 increases by 50% relative to the historical weather conditions (1950 to 1999), under the scenario of Representative Concentration Pathway (RCP) 8.5 (i.e., radiative forcing of 8.5 W⋅m^−2^ in 2100). These unfavorable weather conditions include weakened surface winter northerlies and northwesterlies in the middle troposphere, as well as intensified atmospheric thermal stability in the lower atmosphere. On the basis of downscaling by a regional climate model and under the RCP 4.5 (4.5 W⋅m^−2^ in 2100) warming, the air environment carrying capacity decreases, and the weak ventilation days increase over the entire country except for central China, enhancing the occurrence of haze pollution (116).

A decrease in the area of Arctic sea ice in the preceding autumn and an increase in snowfall in early winter in Eurasia has been attributed as a cause of the severe haze event in the winter 2013 (112), since a change in the northern hemispheric cryosphere leads to poor ventilation conditions. In addition, Wang et al. (111) found high correlation between the observed Arctic sea ice area in autumn and the average winter haze days in eastern China on an interannual scale. Under such a circumstance, continuous warming in the Arctic region is expected to deteriorate the ventilation conditions and increase the frequency and severity of haze pollution in the NCP.

The monsoon circulation, which mainly drives the synoptic weather patterns in East Asia, modulates the PM accumulation and distribution (117–120). The East Asian winter monsoon and the prevailing northwesterly wind play an important role in PM accumulation and removal in northern China (110, 121, 122). The severe haze event in January 2013 has also been associated with the anomalous eastward expansion of Siberian high pressure, while there is no obvious relationship between aerosol optical depth and high pressure intensity (123). The intensity of East Asian winter monsoon exhibits a significant negative correlation with the number of haze days in the central and eastern regions of China, and a weak winter monsoon results in an increase in atmospheric stability and a decrease in vertical diffusion (124). In December 2016, the East Atlantic–West Russia circulation pattern in the middle troposphere strengthened the anticyclone anomaly but suppressed the vertical convection in the NCP, likely responsible for the persistent severe haze formation (125).

Situated on the eastern side of the Tibetan Plateau, northern China is subject to a “harbor” effect of the leeside slope of the plateau under the background westerlies (126). Observations in recent decades reveal that central and eastern China correspond to a “susceptibility zone” of frequent haze formation on a climatological basis. The occurrence of winter haze is positively correlated with the interannual change in the heat source of Tibetan Plateau. The winter monsoon in eastern China is weakened because of the anomalous heat effect, while the downdraft is strengthened, leading to increased stability in the lower troposphere (127).

ENSO also modulates the large-scale circulation in northern China. Recent studies indicated that the strong El Niño event in winter 2015 is responsible for an increased PM_2.5_ concentration by 40 to 80 μg⋅m^−3^ relative to that in winter 2014 (128). During the strong El Niño period, the winter circulation is anomalous and the surface southeasterly wind is significantly strengthened with increasing regional transport in the NCP. There exist significant negative and positive correlations of the number of haze days in winter with sea surface temperature over the western Pacific and eastern equatorial Pacific, respectively (129). On an interdecadal timescale, when Pacific Decadal Oscillation is in a positive phase, the subsidence in central and eastern China is enhanced with increased atmospheric stability, which is conducive to severe haze events (130). Additionally, AMOC has been proposed to influence the strength and position of the westerly jet and constitute a driver of the variability of East Asian winter monsoon (131). Thus, the variation in AMOC may also affect severe haze formation in northern China.

### Sociological Implications.

Haze pollution negatively impacts human health. There has been accumulating evidence that exposure to fine PM causes acute and chronic diseases (21, 22). In addition, UFPs also exert severe health effects, since UFPs are more likely to be deposited in the human pulmonary region and to penetrate into the bloodstream than large particles (132). A recent study revealed that early life exposure to UFPs causes pulmonary immunosuppression (133). Long-term exposure to high levels of PM_2.5_ is estimated to have resulted in 1.1 million deaths in 2015 in China (22). Emissions from coal burning for wintertime residential heating have been suggsted as a main cause for the adverse health impacts (134–137), because coal combustion emits large amounts of toxins, including heavy metals and polycyclic aromatic hydrocarbons. China’s Huai River Policy, which provides free or heavily subsidized coal for wintertime residential heating to cities north of the Huai River, has been found to reduce the life expectancy by 0.64 y for every 10 μg⋅m^−3^ increase in PM_10_ (136).

Visibility in many cities in China has continuously declined since 1990s, at an average of ∼2.1 km per decade (138). Reduced solar radiation associated with haze pollution also impacts the ecosystem. For example, Chameides et al. (139) showed a linear correlation between the crop yield and solar radiation. Tie et al. (140) indicated an up to 28 to 49% reduction of solar irradiance in the four largest crop production regions of China and reduced optimal yields of ∼45% of rice and 75% of wheat growth, leading to 2% reduction in total rice production and 8% reduction in total wheat production in China. Note that there is a large uncertainty in the estimated diffuse solar radiation. In contrast, Yue et al. (141) showed that the ARI effects increase the net primary productivity (NPP) by 0.2 Pg C (5%) through combined diffuse radiation fertilization, reduced canopy temperatures, and reduced evaporation but increased soil moisture. When considering precipitation inhibition from the combined ARI and ACI effects the annual NPP reduces by 0.2 Pg C (4%) which, together with the annual NPP reduction by 0.6 Pg C (14%) from ozone pollution, leads to a net air pollution suppression of 0.8 Pg C (16%) in China.

Air pollution is not regionally isolated and represents a global challenge, considering the emissions and transport of pollutants (142, 143). In particular, anthropogenic emissions are closely related to industrialization, urbanization, and agricultural activities under the global economy (5, 142). Long-range transport of pollutants from Asia has been shown to exert large impacts on global air quality, weather, and climate. A large fraction of anthropogenic emissions of pollutants from China is related to its international trade (142), and nonnegligible amounts of pollutants from China are transported to other countries and affect air pollution and human health worldwide, including the United States (142, 144). In addition, long-range transport of the Asian pollution has been linked to decadal trends of increasing deep convective clouds, precipitation, and transient eddy meridional heat flux, indicative of an intensified winter Pacific storm track (143, 145, 146). An intensified Pacific storm track inevitably influences the global weather and climate (147).

## Conclusion

Severe haze events in northern China can be regarded as synergetic effects from the interactions between anthropogenic emissions and atmospheric processes. These severe haze events occur most frequently in winter, due to seasonally enhanced emissions of pollutants from residential heating (e.g., residential coal combustion and biomass burning), efficient formation of secondary aerosols, and unfavorable meteorological conditions. In addition, climate change (e.g., global warming) may aggravate haze development. The efficient formation of secondary aerosols is attributed to highly elevated concentrations of gaseous precursors, most noticeably VOCs, NO_x_, SO_2_, and NH_3_ emitted from residential heating, traffic sources, and regional agricultural and industrial activities. Currently, available atmospheric chemical mechanisms in the gas and particle phases are insufficient to explain the fine PM evolution from clean to polluted periods, particularly for the formation of SOA and SIA. In addition, the haze events in northern China also provide a unique scientific platform to better understand the formation of secondary aerosols and many aspects of the relevant atmospheric chemical and physical processes.

To improve the understanding of haze formation, refined kinetic and mechanistic data of multiphase chemistry, along with quantification of the aerosol properties, are needed under atmospherically relevant conditions (RH, temperature, and reactant types and abundances). The experimental results are essential not only for atmospheric modeling but also for interpretation and identification of PM in field measurements. Field studies are needed to measure simultaneously gaseous PM precursors and a comprehensive set of the aerosol properties. The field measurements are crucial to providing the temporal and spatial distributions of the gaseous concentrations and PM properties. To achieve a high level of chemical speciation, development of advanced analytical techniques is required, including instruments for identification and quantification of diverse gaseous PM precursors at low levels and particles from the molecular cluster (<1 nm) to submicrometer size ranges. Measurements of the particle density, hygroscopicity, volatility, and optical properties also assist in particle chemical speciation. Furthermore, improved physically based parameterizations of aerosol nucleation and growth developed and validated on the basis of laboratory and field studies are required for incorporation into atmospheric models.

The human health effects of high concentrations of UFPs and high PM_2.5_ mass concentrations need to be carefully evaluated. While many epidemiological studies have emphasized the correlations of the various health syndromes with the PM_2.5_ mass concentration, little is known about the health outcomes of highly elevated levels of UFPs formed from NPF processes under clean conditions. Also, the health effects of other PM properties, including the particle chemical composition and pH, need to be examined. The impacts of climate change on severe haze formation have been recognized in recent years, but a quantitative assessment is lacking, because current global climate models have been unable to resolve many of the subscale atmospheric chemical and physical processes relevant to severe haze formation. The regional and climate impacts of fine PM also need to be assessed to quantify the radiative forcing related to ARI and ACI as well as the feedbacks to atmospheric stability and transport. Furthermore, the impacts of haze pollution on the ecosystem (e.g., crop production and carbon exchange between the biosphere and atmosphere) are not well understood, and future quantitative investigations are needed.

To mitigate haze pollution, effective controls in source emissions of primary PM and secondary PM precursor gases from residential coal combustion, biomass burning, fugitive dust, agricultural activities, and vehicular emissions are essential. Considering the facts of decreasing primary PM levels due to strict regulatory controls by central and local governments but continuously increasing contributions of SIA and SOA in China, further reductions in NO_x_ and SO_2_ and, particularly, effective reductions in the currently much-less-constrained NH_3_ and VOCs are of significant importance (8).

A recent assessment report has established a top research area “to advance the fundamental atmospheric chemistry knowledge that enables predictive capability for the distribution, reactions, and lifetimes of gases and particles” (148). Such a prioritized task is clearly pertinent to improving the understanding of the fundamental mechanisms for severe haze formation in northern China. Specifically, future research is urgently needed to directly address the following scientific questions:

*i*) the mechanisms leading to secondary PM formation, particularly chemical/physical transformation of primary and secondary PM and the associated variations in the PM properties during haze evolution;
*ii*) the interactions and feedback cycles between haze and meteorological/climatic conditions;
*iii*) accurate representations of emissions, chemistry, removal, and transport relevant to fine PM as well as the synergetic effects of these processes in atmospheric models; and
*iv*) quantitative assessment of the climatic conditions, ecosystem, and human health effects of haze pollution.

A better understanding of the aforementioned scientific questions, particularly the interplays between emissions, chemical/physical processes, and meteorology during haze events, are crucial to gain insights into the causes, mechanisms, and trends of haze pollution as well as to assess its impacts on human health, ecosystem, and climate. Such a viewpoint of severe haze formation in China established on the basis of sound science is also critical for improving prediction/forecast of haze pollution, formulating effective regulatory policies by decision makers at the central and local government levels, and raising public awareness of environmental protection. Also, the knowledge and experience in mitigating haze pollution obtained in China can be beneficial and transferred to guide development of effective regulatory policies for other developing countries worldwide.

## Supplementary Material

Supplementary File
