# Supplementary material for: Severe haze in northern China: A synergy of anthropogenic emissions and atmospheric processes
Source: Proc Natl Acad Sci U S A. 2019 Apr 15;116(18):8657–66. doi: 10.1073/pnas.1900125116 (PMC6500134; doi:10.1073/pnas.1900125116)
Supplement: Supplementary File [file pnas.1900125116.sapp.pdf]

PNAS
www.pnas.org

Supporting Information for:

Severe haze in Northern China: A synergy of anthropogenic emissions and atmospheric processes

Zhisheng An, Ru-Jin Huang, Renyi Zhang, Xuexi Tie, Guohui Li, Junji Cao, Weijian Zhou, Zhengguo Shi, Yongming Han, Zhaolin Gu, and Yuemeng Ji

Corresponding author: Zhisheng An

Email: anzs@loess.llqg.ac.cn

This PDF file includes:

Glossary of Acronyms

Figs. S1 to S9

Table S1

References for SI reference citations

www.pnas.org/cgi/doi/10.1073/pnas.1900125116

Glossary of Acronyms

ALWC	Aerosol liquid water content
AMOC	Atlantic meridional overturning circulation
ARI	Aerosol-radiative interaction
ACI	Aerosol-cloud interaction
BC	Black carbon
BTH	Beijing-Tianjin-Hebei
CCN	Cloud condensation nuclei
CNEMC	China National Environmental Monitoring Center
CTMs	Chemical transport models
DU	Dobson unit
EA-WR	East Atlantic-West Russia
EMEP	European Monitoring and Evaluation Programme
ENSO	El Niño and Southern Oscillation
GDP	Gross Domestic Product
HNO_3	Nitric acid
HONO	Nitrous Acid
IN	Ice nuclei
INP	Ice nucleation particles
IPCC	Intergovernmental panel for climate change
LO-OOA	Less oxidized secondary organic aerosol
MO-OOA	More oxidized secondary organic aerosol
NBSC	National Bureau of Statistics of China
NCP	Northern China Plain
NH_4NO_3	Ammonium nitrate
NO_3	Nitrate radical
NO_x	Nitrogen oxides
NPF	New particle formation
NPP	Net primary productivity
OA	Organic aerosol
OH	Hydroxyl radical
OM	Organic matter
OMI	Ozone Monitoring Instrument
OVOCs	Oxygenated volatile organic compounds
O_x	Odd oxygen concentrations (O_x= O_3+NO_2)
PAHs	Polycyclic aromatic hydrocarbons
PBL	Planetary boundary layer
PDO	Pacific Decadal Oscillations
PM	Particulate matter
$PM_{2.5}$	Particles with the aerodynamic diameter smaller than 2.5 μm
PM_{10}	Particles with the aerodynamic diameter smaller than 10 μm
ppb	Parts per billion
ppt	Parts per trillion
POA	Primary organic aerosol
RH	Relative humidity

RCP	Representative Concentration Pathway
SIA	Secondary inorganic aerosol
SOA	Secondary organic aerosol
SSA	Single-scattering albedo
SST	Sea surface temperature
VOC(s)	Volatile organic compound(s)
WHO	World Health Organization
WMO	World Meteorological Organization
WRF-CHEM	Weather Research and Forecasting model coupled to Chemistry

Retrieving historical $PM_{2.5}$ concentrations

Daily atmospheric visibility and $PM_{2.5}$ mass concentrations from 20 observation sites in the North China Plain (NCP) were used to retrieve the historical $PM_{2.5}$ concentrations. Note that the 20 observation sites are shown in Fig. S1 and S2. The visibility data for the period of 1973–2017 were obtained from the U.S. National Climatic Data Center (NCDC), while $PM_{2.5}$ mass concentrations for the period of 2013–2017 were collected from the China National Environmental Monitoring Center (CNEMC). A linear regression was established between the $PM_{2.5}$ mass concentration and the logarithm of visibility using the data during 2013–2015, and the correlation coefficients (R^2) ranged from 0.36–0.72 (Fig. S1). To evaluate the reliability of the linear regression model, the visibility data during 2016–2017 were used to calculate the mass concentrations of $PM_{2.5}$, and the predicted $PM_{2.5}$ values were correlated well with the measured ones (R^2 = 0.45–0.69, Fig. S2), suggesting that the visibility can be a good surrogate for $PM_{2.5}$. Therefore, based on these linear regression formulas in Fig. S1, the long-term range of visibility data was used to retrieve the historical $PM_{2.5}$ mass concentrations in the NCP. Note that the trough of $PM_{2.5}$ from 2007-2012 (See Fig. 1 in the main text) is consistent with a previous study retrieved by satellite aerosol optical depth (AOD) data, which could be attributed to the implement of the "Energy Conservation and Emissions Reduction" policy from 2006 (1).

WRF-CHEM model and configuration

The WRF-CHEM model used in the study is developed by Li et al. (2-5) at the Molina Center for Energy and the Environment, with a new flexible gas phase chemical module and the CMAQ aerosol module developed by US EPA. The aerosol component of the Community Multiscale Air Quality (CMAQ) model is designed to be an efficient and economical depiction of aerosol dynamics in the atmosphere (6). The particle size distribution in the study is represented as the superposition of three lognormal subdistributions, called modes, which includes the processes of coagulation, particle growth by the addition of mass, and new particle formation. Following the work of Kulmala et al. (7), the new particle production rate presented here is calculated as a parameterized function of temperature, relative humidity, and the vapor-phase H_2SO_4 concentration due to binary nucleation of H_2SO_4 and H_2O vapor, and the new particles are assumed to be 2.0 nm diameter. The wet deposition follows the method used in the CMAQ and the surface deposition of chemical species is parameterized following Wesely (8). The photolysis

rates are calculated using the FTUV (3, 9), in which the effects of aerosols and clouds on photolysis are considered.

The inorganic aerosols are predicted in the WRF-CHEM model using ISORROPIA Version 1.7 (10). The secondary organic aerosol (SOA) formation is calculated using a non-traditional SOA module. The volatility basis-set (VBS) modeling method is used in the module, assuming that primary organic components are semi-volatile and photochemically reactive and are distributed in logarithmically spaced volatility bins. Detailed information about the volatility basis-set approach can be found in Li et al. (4). The SOA formation from glyoxal and methylglyoxal in this study is parameterized as a first-order irreversible uptake by aerosol particles and cloud droplets, with a reactive uptake coefficient of 3.7×10^{-3} for glyoxal and methylglyoxal (11-13).

A persistent air pollution episode from 04 December 2015 to 27 December 2015 in the NCP is simulated using the WRF-CHEM model (Table S1). The WRF-CHEM model adopts one grid with horizontal resolution of 12 km and 35 sigma levels in the vertical direction, and the grid cells used for the domain are 400 × 400. The physical parameterizations include the microphysics scheme of Hong and Lim (14), the Mellor, Yamada, and Janjic (MYJ) turbulent kinetic energy (TKE) planetary boundary layer scheme (15), the Unified Noah land-surface model (16), the rapid radiative transfer model (RRTM) long wave radiation scheme (17) and the Goddard shortwave parameterization (18, 19). The National Centers for Environmental Prediction (NCEP) 1° × 1° reanalysis data are used to obtain the meteorological initial and boundary conditions, and the meteorological simulations are not nudged in the study. The chemical initial and boundary conditions are interpolated from the 6 h output of MOZART (20). The spin-up time of the WRF-CHEM model is 28 h. The SAPRC-99 (Statewide Air Pollution Research Center, version 1999) chemical mechanism is used in the present study.

The anthropogenic emissions are developed by Zhang et al. (21), which is based on the 2012 emission inventory, including contributions from agriculture, industry, power generation, residential, and transportation sources. In the SAPRC-99, aromatics are lumped into ARO1 and ARO2. ARO1 mainly includes toluene, benzene, ethylbenzene, and other aromatics with reaction rate with OH (k_{OH}) less than 2×10^4 ppm^{-1} min^{-1}. ARO2 includes xylene, trimethylbenzene, and other aromatics with k_{OH} greater than 2×10^4 ppm^{-1} min^{-1}. Additionally, biogenic VOCs also play a considerable role in the ozone production (22), and monoterpenes and isoprene are the main biogenic VOCs in the SAPRC-99 chemical mechanism. The biogenic emissions are calculated

online using the MEGAN (Model of Emissions of Gases and Aerosol from Nature) model developed by Guenther et al (23).

Table S1 WRF-CHEM model configurations.

Region	East Asia
Simulation period	04 December 2015 to 27 December 2015
Domain size	400 × 400
Domain center	35°N, 114°E
Horizontal resolution	12 km × 12 km
Vertical resolution	35 vertical levels with a stretched vertical grid with spacing ranging from 30 m near the surface, to 500 m at 2.5 km, and to 1 km above 14 km
Microphysics scheme	WSM 6-class graupel scheme (14)
Cumulus scheme	Grell-Devenyi ensemble scheme (24)
Boundary layer scheme	MYJ TKE scheme (15)
Surface layer scheme	MYJ surface scheme (15)
Land-surface scheme	Unified Noah land-surface model (16)
Longwave radiation scheme	Goddard longwave scheme (25)
Shortwave radiation scheme	Goddard shortwave scheme (19)
Meteorological boundary and initial conditions	NCEP 1°×1° reanalysis data
Chemical initial and boundary conditions	MOZART 6-hour output (20)
Anthropogenic emission inventory	Developed by Zhang et al. (21) and Li et al. (26, 27), 2012 base year, and SAPRC-99 chemical mechanism
Biogenic emission inventory	Online MEGAN model developed by Guenther et al. (23)

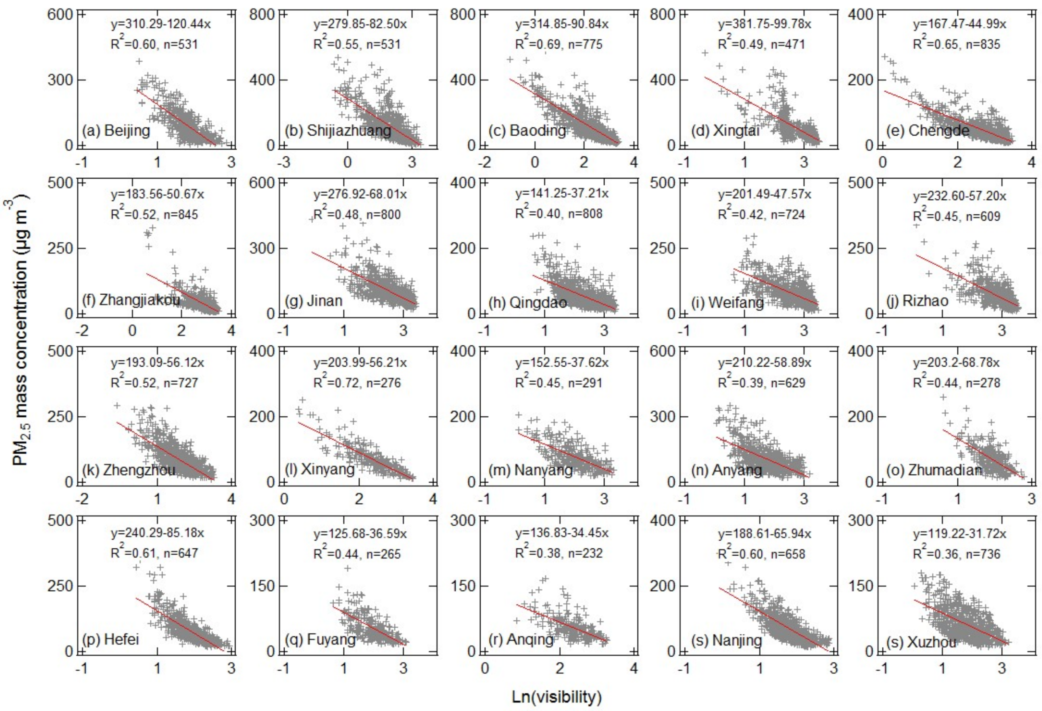

Fig. S1. Correlations between $PM_{2.5}$ mass concentration and logarithm of visibility at 20 observation sites in the NCP during 2013–2015.

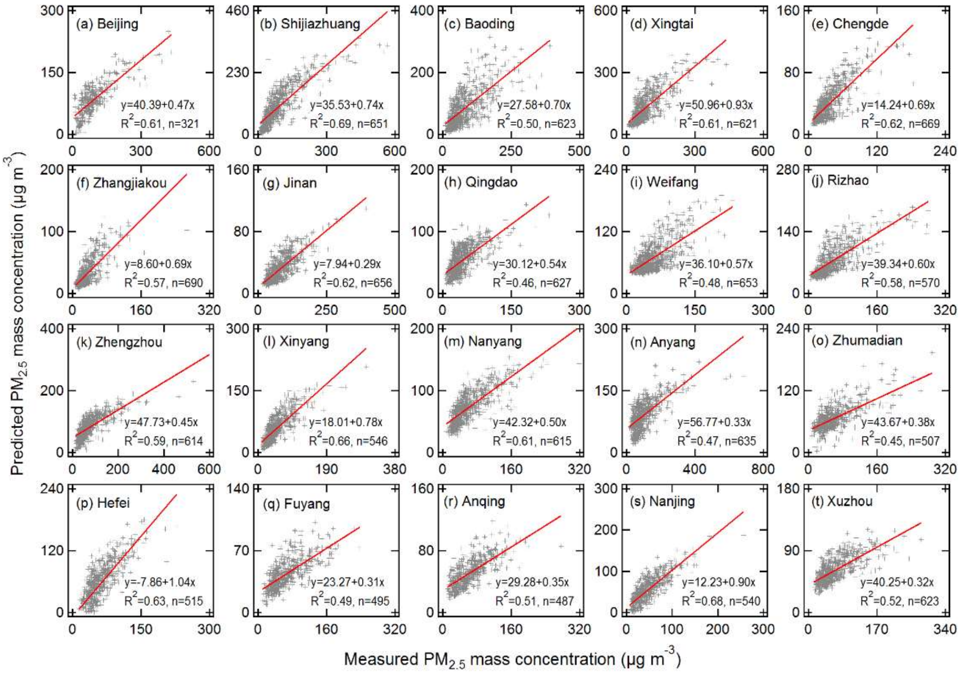

Fig. S2. Correlations between predicated and measured $PM_{2.5}$ mass concentrations.

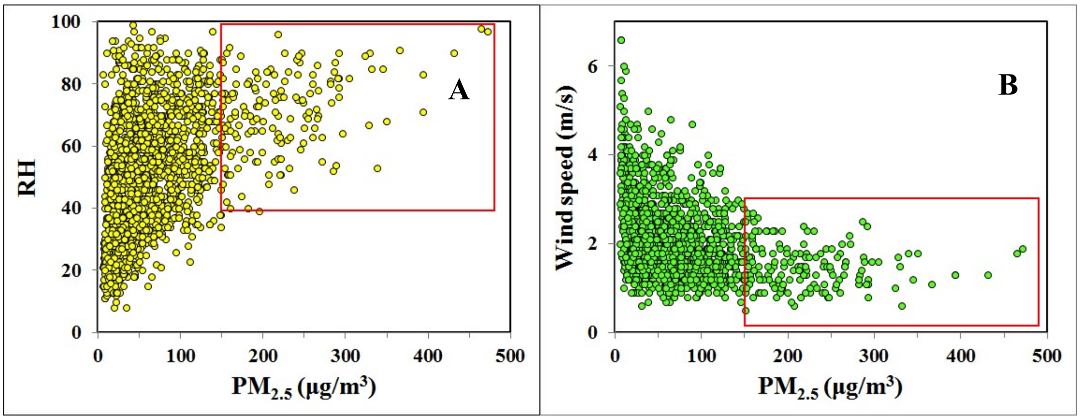

Fig. S3. The relationship between daily $PM_{2.5}$ concentration and daily RH (A) and between daily $PM_{2.5}$ concentration and daily wind speed (B) measured in Beijing from 01 April 2013 to 11 December 2017. Data in the red rectangular frames indicate that high $PM_{2.5}$ concentrations ($\geq$ 150 μg m^{-3}) are associated with high RH and low wind speed. Note that the $PM_{2.5}$ data were from the China National Environmental Monitoring Center (CNEMC), and RH and wind speed from China Meteorological Administration.

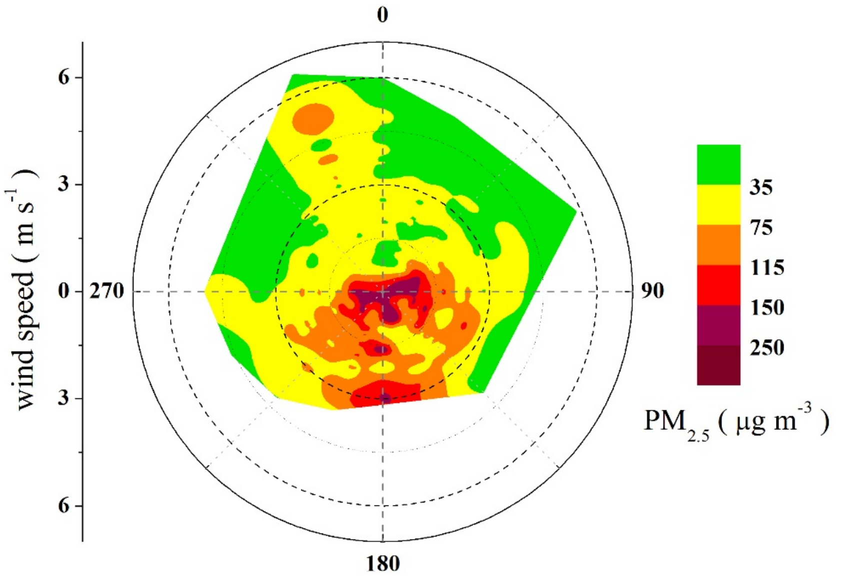

Fig. S4. The daily $PM_{2.5}$ concentration, wind speed and wind direction measured in Beijing from 01 April 2013 to 11 December 2017. Note that the $PM_{2.5}$ data were from the China National Environmental Monitoring Center (CNEMC), and wind speed from China Meteorological Administration.

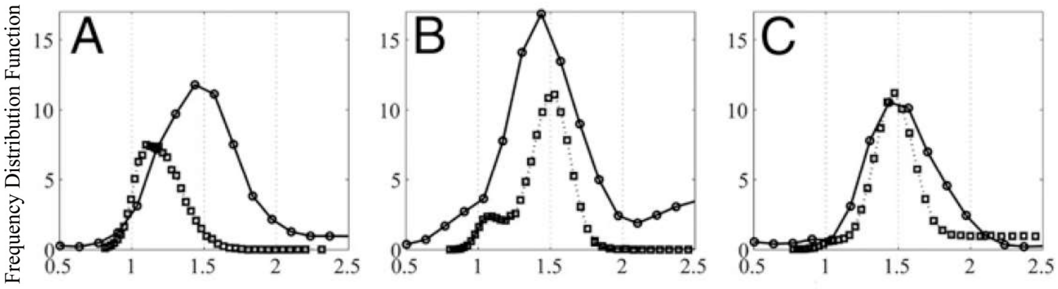

Fig. S5. Particle hygroscopicity and density during the clean, transition, and polluted periods for the 25–29 September and 2–7 October episodes. (A−C) Effective density (solid line, circles) and hygroscopicity (dashed line, squares) for 46-nm (A), 97-nm (B), and 240-nm (C) particles at 1500 h on 25 September, 1200 h on 27 September, and 1800 h on 28 September, respectively. (Reprinted from ref. 28).

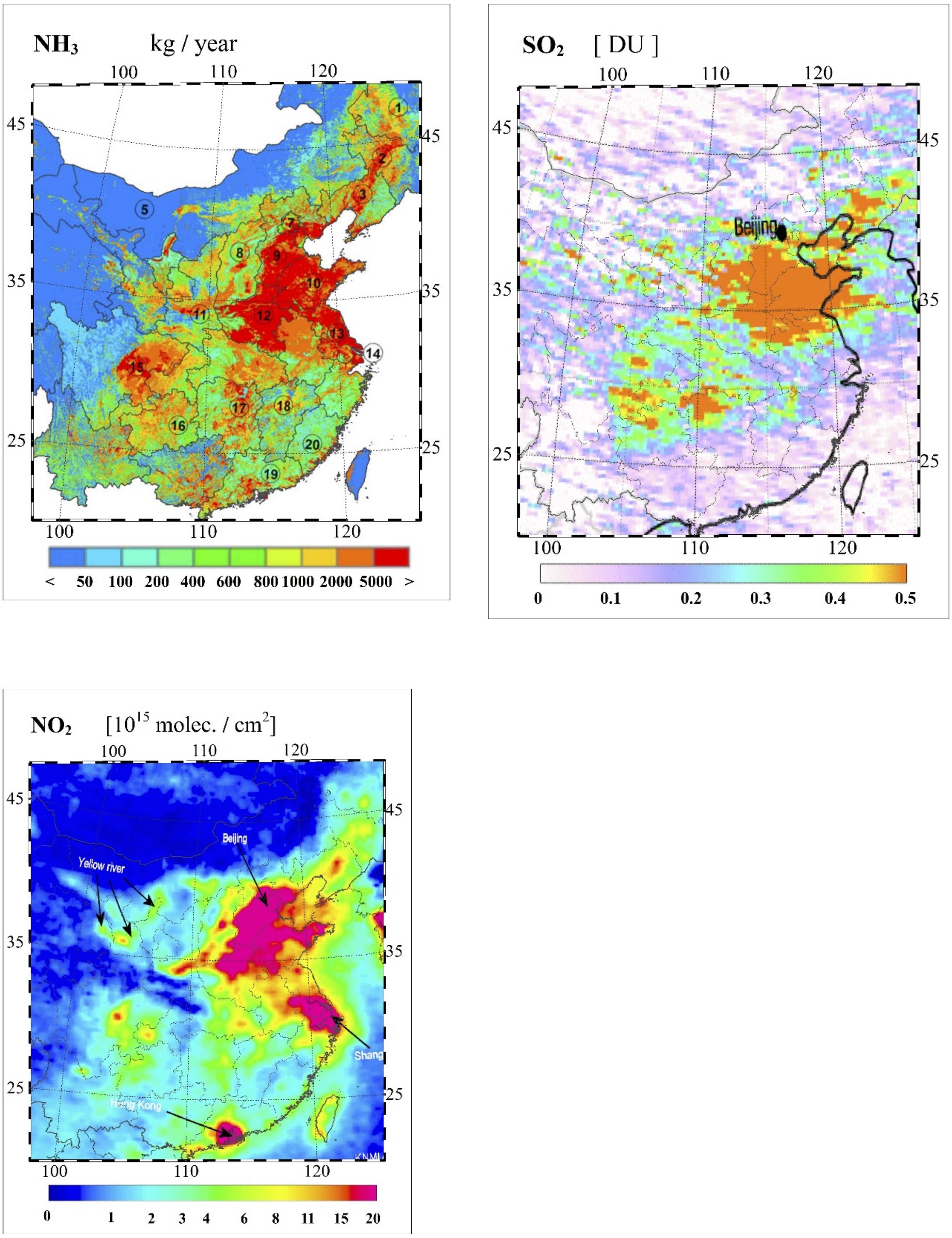

Fig. S6. The spatial distribution of NH_3, SO_2 and NO_2 in China, showing the overlapping of agricultural NH_3 with industrial SO_2 and NO_2. Note that NH_3 data were from Huang et al. (reprinted with permission from ref. 29), SO_2 data from SCIAMACHY (https://earth.esa.int), and NO_2 data from KNMI/IASB/ESA (Royal Netherlands Meteorological Institute, KNMI).

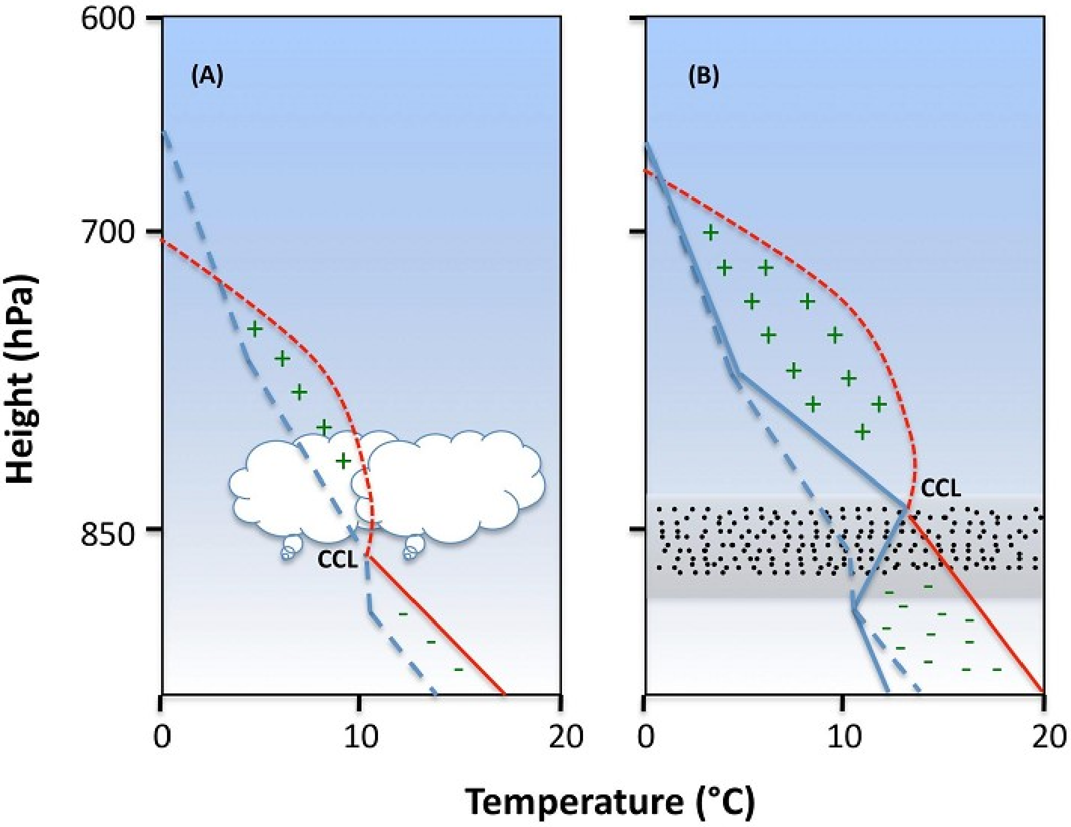

Fig. S7. Schematic depiction of the atmospheric effects of absorbing aerosols on convection and cloud formation: (*A*) without and (*B*) with the presence of scattering and absorbing aerosols in the PBL. The dashed and solid blue lines correspond to the vertical temperature profiles in the absence and presence of the scattering and absorbing aerosol layer, respectively, and the solid and dashed red lines denote the dry and moist adiabats, respectively. The reduced surface temperature and the increased temperature aloft due to ARI under the polluted conditions lead to a larger negative energy with convective inhibition (-) and a higher convection condensation level (CCL), but induces a larger convective available potential energy (+) above CCL. (Reprinted from ref. 30. Copyright (2013), with permission from Elsevier.).

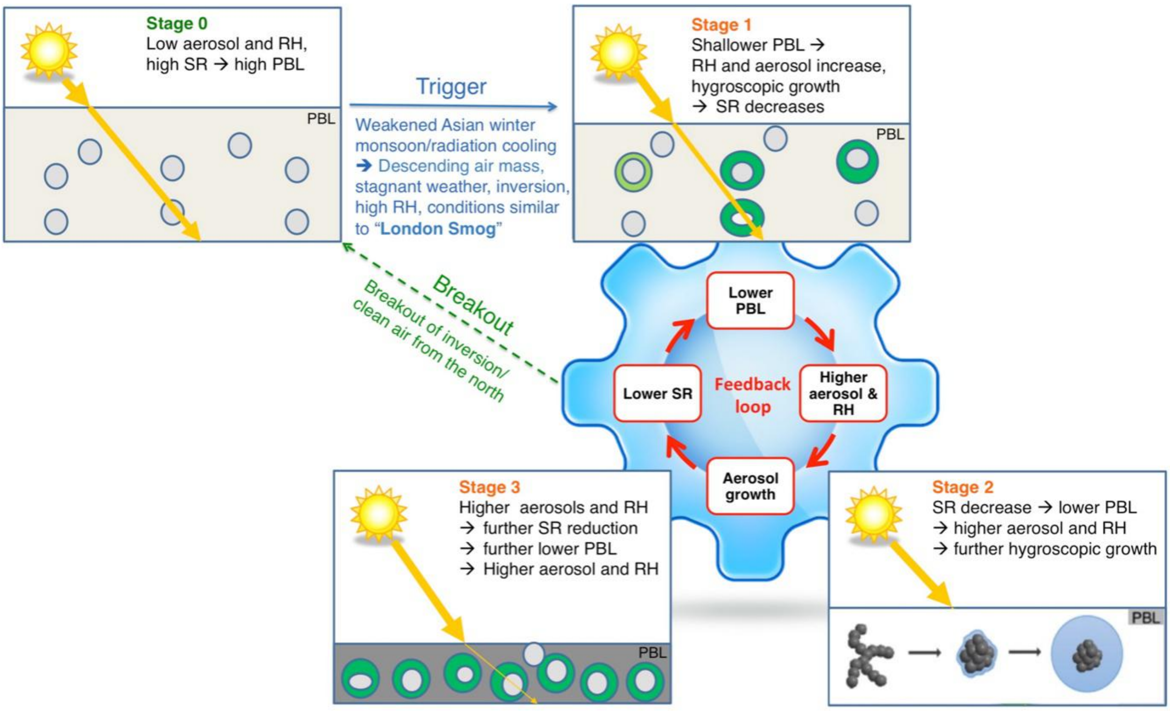

Fig. S8. Feedback mechanism amplifies the formation of severe haze events by aerosol-water-radiation interaction (modified from refs. 31 and 32).

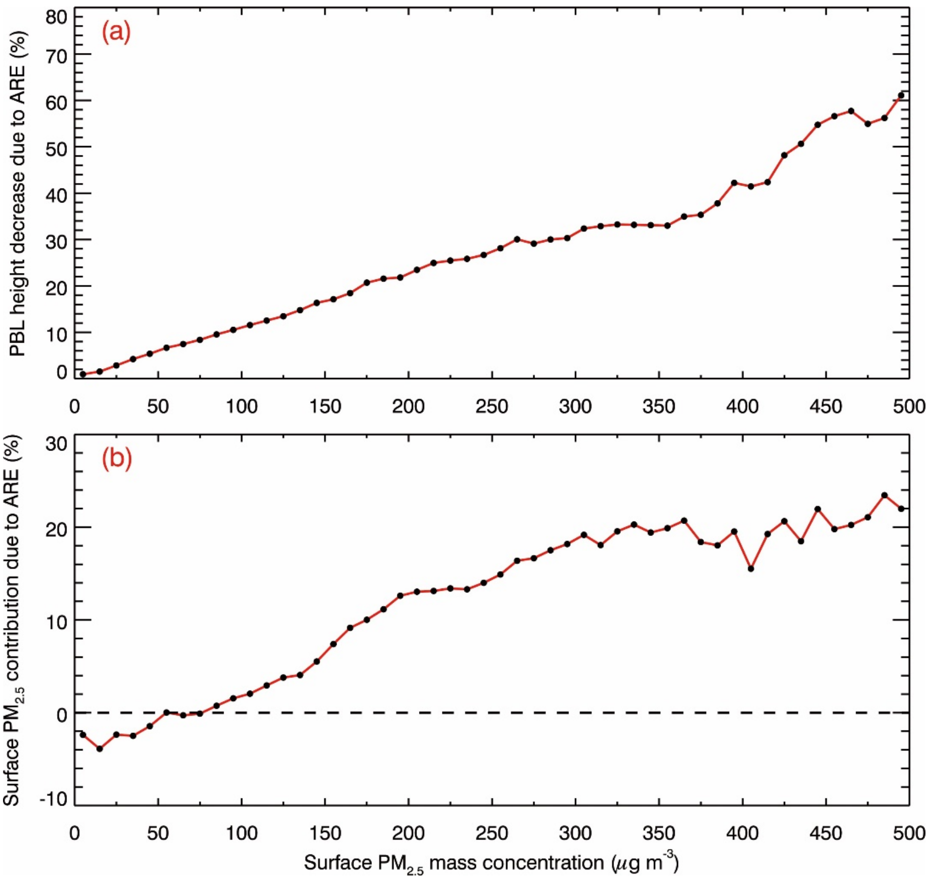

Fig. S9. ARI induces PBL height decrease (A) and surface $PM_{2.5}$ contribution (B) as a function of the surface $PM_{2.5}$ mass concentration in the NCP during the PBL height peak time from 04 to 27 December 2015, simulated by WRF-CHEM.

References

1. Ma ZW, et al. (2016) Satellite-Based Spatiotemporal Trends in $PM_{2.5}$ Concentrations: China, 2004–2013. *Environ Health Persp* 124:184-192.
2. Li GH, et al. (2010) Impacts of HONO sources on the photochemistry in Mexico City during the MCMA-2006/MILAGO Campaign. *Atmos Chem Phys* 10:6551-6567.
3. Li GH, Bei NF, Tie XX, Molina LT (2011a) Aerosol effects on the photochemistry in Mexico City during MCMA-2006/MILAGRO campaign. *Atmos Chem Phys* 11:5169-5182.
4. Li GH, et al. (2011b) Simulations of organic aerosol concentrations in Mexico City using the WRF-CHEM model during the MCMA-2006/MILAGRO campaign. *Atmos Chem Phys* 11:3789-3809.
5. Li GH, Lei W, Bei NF, Molina LT (2012) Contribution of garbage burning to chloride and $PM_{2.5}$ in Mexico City. *Atmos Chem Phys* 12:8751-8761.
6. Binkowski FS, Roselle SJ (2003) Models-3 Community Multiscale Air Quality (CMAQ) model aerosol component: 1. Model description. *J Geophys Res* 108(D6): 4183, doi:10.1029/2001JD001409, 2003
7. Kulmala M, Laaksonen A, Pirjola L (1998) Parameterizations for sulfuric acid/water nucleation rates. *J Geophys Res Atmos* 103: 8301– 8307.
8. Wesely ML (1989) Parameterization of surface resistances to gaseous dry deposition in regional-scale numerical models. *Atmos Environ* 23:1293-1304.
9. Li GH, Zhang RY, Fan JW, Tie XX (2005) Impacts of black carbon aerosol on photolysis and ozone. *J Geophys Res* 110:D23206, doi:10.1029/2005jd005898, 2005.
10. Nenes A, Pilinis C, Pandis SN (1998) ISORROPIA: A New thermodynamic equilibrium model for multiphase multicomponent inorganic aerosols. *Aquat Geochem* 4(1): 123-152.
11. Zhao J, Levitt NP, Zhang RY, Chen JM (2006) Heterogeneous reactions of methylglyoxal in acidic media: implications for secondary organic aerosol formation. *Environ Sci Technol* 40:7682–7687.
12. Volkamer R, et al. (2007) A Missing Sink for Gas-Phase Glyoxal in Mexico City:

Formation of Secondary Organic Aerosol. *Geophys Res Lett* 34:L19807, doi:10.1029/2007GL030752, 2007.

13. Gomez ME, Lin Y, Guo S, Zhang RY (2015) Heterogeneous Chemistry of Glyoxal on Acidic Solutions. An Oligomerization Pathway for Secondary Organic Aerosol Formation. *J Phys Chem A* 119:4457-4463.
14. Hong SY, Lim J-OJ (2006) The WRF Single-Moment 6-Class Microphysics Scheme (WSM6). *J Korean Meteorol Soc* 42:129-151.
15. Janjić ZI (2002) Nonsingular Implementation of the Mellor–Yamada Level 2.5 Scheme in the NCEP Meso Model. *Ncep Office Note*, 436.
16. Chen F, Dudhia J (2001) Coupling an advanced land surface-hydrology model with the Penn State-NCAR MM5 modeling system. Part I: Model implementation and sensitivity. *Mon Weather Rev* 129: 569-585.
17. Mlawer EJ, Taubman SJ, Brown PD, Iacono MJ, Clough SA (1997) Radiative transfer for inhomogeneous atmospheres: RRTM, a validated correlated-k model for the longwave. *J Geophys Res Atmos* 102:16,663-16,682.
18. Suarex MJ, Chou MD (1994) Technical report series on global modeling and data assimilation. *Volume 3: An efficient thermal infrared radiation parameterization for use in general circulation models* 3:1603-1609.
19. Chou MD, Suarez MJ (1999) A solar radiation parameterization for atmospheric studies. *NASA TM-104606, Nasa Tech memo,* 15.
20. Horowitz LW, et al. A global simulation of tropospheric ozone and related tracers: Description and evaluation of MOZART, version 2. *J Geophys Res Atmos* 108(D24):4784, doi:10.1029/2002jd002853, 2003.
21. Zhang Q, et al. (2009) Asian emissions in 2006 for the NASA INTEX-B mission. *Atmos Chem Phys* 9:5131-5153.
22. Li GH, Zhang RY, Fan JW, Tie XX (2007) Impacts of biogenic emissions on photochemical ozone production in Houston, Texas. *J Geophys Res* 112:D10309, doi:10.1029/2006jd007924, 2007.

23. Guenther A, et al. Estimates of global terrestrial isoprene emissions using MEGAN (Model of Emissions of Gases and Aerosols from Nature). *Atmos Chem Phys* 6:3181-3210.

24. Grell GA, Devenyi D (2002) A generalized approach to parameterizing convection combining ensemble and data assimilation techniques. *Geophys Res Lett* 29(14):1693, doi:10.1029/2002GL015311, 2002.

25. Chou MD, Suarez MJ, Liang XZ, Yan MH, Cote C (2001) A Thermal Infrared Radiation Parameterization for Atmospheric Studies. *NASA/TM-2001-104606, Vol. 19.*

26. Li GH, et al. (2017a) A possible pathway for rapid growth of sulfate during haze days in China. *Atmos Chem Phys* 17:3301-3316.

27. Li G, et al. (2017b) Widespread and persistent ozone pollution in eastern China during the non-winter season of 2015: observations and source attributions**.** *Atmos Chem Phys* 17**:**27 59-2774.

28. Guo S, et al. (2014) Elucidating severe urban haze formation in China. *Proc Natl Acad Sci USA* 111(49):17373-17378.

29. Huang X, et al. (2012) A high-resolution ammonia emission inventory in China. Global Biogeochem Cy, 26, GB1030, doi:1010.1029/2011GB004161.

30. Wang Y, Khalizov A, Levy M, Zhang R (2013) New Directions: Light absorbing aerosols and their atmospheric impacts. *Atmos Environ* 81(4): 713-715.

31. Tie XX, et al. (2017) Severe Pollution in China Amplified by Atmospheric Moisture. *Sci Rep* 7:15760, doi:10.1038/s41598-017-15909-1.

32. Peng J, et al. (2016) Markedly enhanced absorption and direct radiative forcing of black carbon under polluted urban environments. Proc Natl Acad Sci USA 113:4266-4271.
